# Integrating Rough-and-Tumble Play in Martial Arts: A Practitioner’s Model

**DOI:** 10.3389/fpsyg.2021.731000

**Published:** 2021-09-30

**Authors:** Tony Blomqvist Mickelsson, Pilo Stylin

**Affiliations:** ^1^ Södertörn University, Huddinge, Sweden; ^2^ Swedish Budo and Martial Arts Confederation, Stockholm, Sweden

**Keywords:** martial arts, rough-and-tumble play, self-regulation, empathy, prosocial

## Abstract

This paper introduces a model that explains psychosocial development by embedding the developmental concept of rough-and-tumble play (RTP) into the contextual settings of martial arts (MA). Current sport-for-change literature relies on theories that address contextual factors surrounding sport but agrees that sport in itself does not facilitate developmental outcomes. In contemporary times where western societies invest substantial resources in sport programs for their psychosocial contribution, this becomes problematic. If the contextual factors surrounding sport are exclusively what produce developmental outcomes, what is the rationale for investing resources in *sport* specifically? We challenge this idea and argue that although contextual factors are important to any social phenomena, the developmental outcomes from sport can also be traced to the corporeal domain in sport. To date, we have lacked the theoretical lenses to articulate this. The developmental concept of RTP emphasizes how “play fighting” between consenting parties stimulates psychosocial growth through its demand for self-regulation and control when “play fighting” with peers. In short, RTP demands that individuals maintain a self-regulated mode of fighting and is contingent on a give-and-take relationship to maintain enjoyment. RTP can thus foster empathy and prosocial behavior and has strong social bonding implications. However, such play can also escalate. A fitting setting to be considered as moderated RTP is MA because of its resemblance to RTP, and its inherent philosophical features, which emphasizes self-regulation, empathy, and prosocial behavior. This paper outlines what constitutes high-quality RTP in a MA context and how this relates to developmental outcomes. By doing so, we present a practitioner’s framework in which practitioners, social workers, and physical educators can explain how MA, and not merely contextual factors, contributes toward developmental outcomes. In a time where sport is becoming increasingly politicized and used as a social intervention, it too becomes imperative to account for why *sport*, and in this case, MA, is suitable to such ends.

## Introduction

This article is a transdisciplinary collaboration between the Swedish Budo and Martial Arts Federation (SBMAF) and the local institution. We jointly set out to propose an applied model that explains how MA can contribute to psychosocial outcomes. Specifically, we link the theoretical underpinnings of RTP and embed them within a MA context. This is performed as part of the backdrop of the sport-for-change research that (1) has been accused of being poorly theorized and (2) rejects the notion that sport in itself contributes toward developmental goals. Instead, contemporary theories propose that sport serves as a platform in which contextual factors facilitate developmental outcomes. This becomes troubling for a variety of reasons. First, if we reject the idea that *sport* specifically is important for developmental outcomes, but only contextual factors, why should sport specifically continue to be promoted and funded to the current extent? Second, this becomes even more curious considering some research shows equally or even stronger psychosocial effects of other leisure activities.

Instead, we propose that the corporeal experience of certain sports can, under decent circumstances, directly contribute to psychosocial development. However, we need to examine this through a different theoretical lens. Instead of exclusively adhering to the explanatory power of contextual factors, we utilize the concept of RTP. RTP stems from developmental psychology and is concerned with how “play fighting” can facilitate developmental outcomes. This entails a playful collaboration contingent on consent, self-regulation, and empathy between two or more individuals. However, play fighting in its natural setting is unsupervised and may escalate. Thus, we suggest that RTP can serve as an analytical framework when embedded within a MA context. The principal affinities between RTP and MA are plentiful and strong, making this theoretical concept especially fruitful.

We propose a narrower but directly applicable theoretical and practical framework for practitioners engaging in the sport-for-change field. This is important given the poor conceptualizations and vaguely defined outcomes that have so far characterized the field, and which have rendered the evidence for the psychosocial effect of sport in itself challenging to explain. Secondly, it justifies why aspects of *sport* are important, and not only the contextual factors surrounding it.

The paper proceeds as follows. Firstly, we position ourselves within the MA research and practitioners’ community and clarify the background that made this paper possible. Secondly, we account for the current state of sport, and the theories that explain the change that has dominated the field. Thirdly, we account for RTP, its contributions, and its current evidence base. Subsequently, we link how this evidence base fits into the frames of MA, where RTP can thrive, be moderated, and governed in order to actively seek developmental outcomes. Finally, we present an ecological model and finish by discussing the model and its limitations.

## Background

### Positioning Ourselves in the Field

This paper is written based on a long commitment to MA, both from a practitioner and academic perspective. Making this commitment transparent, we admit our bias and passion for MA but also make our wealth of experience clear along with the inherent potential for the psychosocial effect of MA that both authors have witnessed. Through this paper, we hope to contribute to theoretical advancements in the research community and directly inform future models that bear direct implications for SBMAF’s (and other organizations’) practices. These practices already exist but can be considered as in their infancy. Some of the practices intended for development are education for leaders and more scientifically grounded models to base their work on. Thus, this paper aims to bridge the practitioner – academic gap, with direct implications for social work practice and theory.

The first author’s research interest includes MA’s sociopsychological contribution (e.g., Blomqvist Mickelsson, 2020, 2021; Mickelsson Blomqvist and Hansson, 2021). This research interest originates from 15years of MA practice. This includes training and competing as an amateur and a brief stint as a professional, involving various MAs, including Brazilian jiu-jitsu, mixed martial arts, sambo, and Thai boxing. Notably, the first author has maintained a full-time profession as a coach throughout his mid-twenties, coaching both adults and young people. As with most MA trainers, this has come with an implicit responsibility in governing (mostly) young people’s psychosocial health in the MA environment.

The second author represents SBMAF. SBMAF is one of a few global confederations that serves as a national umbrella confederation, governing 30 different MAs in Sweden. In general, Swedish sport is held in high regard, with an annual state funding of two billion SEK a year (approximately 200,000 USD), contributing to the development of Swedish sport. This prioritization of sport in Sweden is reflected in its high numbers of organizations and members (Fahlén and Stenling, 2016). The second author is a sports consultant, a qualified elite coach, and shoulders the responsibility for developing the youth sports section in SBMAF. Complementing this experience from SBMAF, the second author also has 20years of coaching experience in MA, and 10years of being an elite competitor along with running his MA club.

It should be mentioned that, while we uniformly agree that MA is a potent vehicle for psychosocial change, both authors have witnessed the detriments of MA, including eating disorders, social vulnerability, and more (Blomqvist Mickelsson et al., 2020; Mickelsson Blomqvist and Hansson, 2021). Nevertheless, as the backdrop to this experience, it has informed the model in conjunction with the psychosocial literature on MA and developmental psychology.

### The Current State of Sport and Developmental Theory

There is a strong notion that sport contributes to various developmental outcomes; some of the most touted claims have been psychosocial development and adequate socialization. However, these claims are only partially substantiated as evidenced by recent longitudinal research. For example, Neville et al. (2021) found that organized sport was associated with a decrease in troubled behavior among boys, but no significant decreases were found for girls; neither was any prosocial effect due to sport present. Likewise, Lundkvist et al. (2020) detected an effect on sport’s capacity to promote friendship, but only to a (very) small degree. Growing as a field of its own, sport for development (SFD) has emerged as a response to the increasing use of SFD outcomes. However, despite the increasing interest in SFD, which is both visible in academia and among other stakeholders, theory in sport for change remains underdeveloped (Coalter, 2015).

In a recent comprehensive review of 437 studies focused on sports developmental outcomes, the most dominating theoretical frameworks were positive youth development (PYD) and social capital (Schulenkorf et al., 2016). Soccer was shown to be the most utilized sport, primarily because of its popularity. Another review aimed at life-skill acquisition in sport among socially vulnerable youths found similar results where PYD principles also dominated the bulk of included studies (Hermens et al., 2017). The transfer of being taught life skills in sport and bringing these to the outside world has been a central point for sport researchers, which is intertwined in PYD theory (Jacobs and Wright, 2018). Stemming from developmental sciences that merged with positive psychology, PYD has been adapted to sport, characterized, for example, through the “4Cs” (competence, confidence, connection, and character) approach by Vierimaa et al. (2012). In short, PYD theory focuses on contextual factors that facilitate healthy personal development. These include factors, such as the coach (knowledge, relationship with students, etc.), peers, parents, and intrapersonal factors (e.g., moral), the learning climate (e.g., mastery oriented *vs.* goal oriented), to mention a few. Hermens et al.’s (2017) review reveals that many studies implement PYD in a context where youths are also actively taught personal and social responsibility. Contemporarily, PYD (and soccer) continues to dominate the theoretical domain of the field.

On the other hand, social capital theory in sport mainly references Putnam’s (2000) ideas. Social capital theory in sport advocates that sport can provide an arena where individuals with both shared and diverse backgrounds can meet and interact on equal grounds because of sport’s “universal language.” In Putnam’s (2000) terminology, this can lead to bonding, which refers to increasingly stronger social ties between individuals that share a common denominator (most often ethnic). In addition, it can lead to bridging, which mainly refers to the creation of inter-ethnic friendship. Ultimately, the expansion of social capital has more significant societal effects, such as political trust and civic engagement. Contrasted with PYD, social capital emphasizes ideas, such as group membership, collective identity, and similar concepts in a sociological spirit.

Social capital theory also views sport as an arena that can potentially connect people much like other areas in civil society. Sport is thus not a socialization vehicle *per se* but provides a context in which socialization may occur. Yet, it is also evident that bridging rarely occurs (Walseth, 2016; Lundkvist et al., 2020), which also points to the need to re-organize how we think about sport as an arena for (inter-ethnic) friendship.

Positive youth development and social capital theory consist of generic factors readily available to be implemented in a host of contexts in and out of sport. Indeed, there is now a general agreement that sport in *itself* does not lead to developmental outcomes but rather the context, such as coaches, peers, and philosophies (Holt et al., 2020). This implies that any group membership can facilitate developmental outcomes under decent conditions. The result is that the probable unique feature of sport is its physicality. In other words, healthy contextual factors surrounding *any* activity will most likely produce developmental outcomes; this renders the activity of sport in itself less significant. In reviewing, early research on sport Reppucci (1987, p.10) correctly stated that as:

*“…there is little, if any, valid evidence that participation in sport is an important or essential element of the socialization process, or that involvement in sport teaches or results in the learning of specific outcomes that might not be learned in other social milieu.*”

Delaney and Keaney (2005) analyzed panel data from the European social survey on sport participation and psychosocial effects. The authors also referred to similar findings on general membership in organizations and concluded that it might not be sport *per se* but instead group membership that plays a key role. More worrying is that in a random sample of Norwegians, sport generally had a *weaker* social effect than membership of other voluntary organizations (Seippel, 2006). Other research also shows that the psychosocial effect of sport compared to other activities is not as astonishing as policymakers usually believe (Bishop et al., 2002; Molinuevo et al., 2010; Kwak et al., 2018).

Seippel (2006) also explicates this paper’s concern; nothing supports the supposition that sport specifically is more suitable than other activities to support developmental outcomes. Many believe that sport produces social capital, respect, and psychosocial development, but exactly how is *sport* responsible for this, and not merely the contextual factors surrounding it? This is a focal point of the current paper. Although we acknowledge that contextual factors are important (to any social phenomena), we challenge the idea that sport in itself is not viewed as a catalyst for psychosocial development. As has been argued, this has political implications.

We argue that the current framework will allow practitioners to address and govern the change they aim to produce. We turn to another branch of developmental science to address how we can understand sport as not merely an empty mediator enriched with contextual factors, and instead as *the* catalyst for psychosocial development.

### Rough-and-Tumble Play

RTP is a concept that entails social playing that has been coined “play fighting” (Pellis and Pellis, 2007). In further defining RTP, it has been argued to be characterized by physical and vigorous behaviors, e.g., chasing, jumping, and play fighting accompanied by positive emotions between the parties involved (Pellegrini, 1988). What follows is that RTP mimics fighting, but it needs to involve consenting parties (DiPietro, 1981).

Consequently, RTP also resembles general “pretend play,” which is of educational use for children as they learn to self-regulate, act accordingly to different social situations, and engage in social negotiation with one another (Lillard et al., 2013). StGeorge and Fletcher (2019, p.1) posited that RTP “…appears to function for children as a compelling learning environment for social and emotional skills.” RTP goes beyond the effects at a mere physical and motor level, although these are also important. According to Peterson and Flanders’ (2005) model, RTP demands the child to engage and refine self-regulatory abilities because of the exciting and intense elements, but also because RTP can only be sustained if both partners are willing to engage. In terms of play, it becomes crucial that both children are inclined to play at an equal level. That is, if a physically more powerful child deprives the counterpart’s ability to actively engage in play due to complete dominance, the play is likely to stop. As such, children are allowed to adjust their intensity and power according to the other child’s ability and need (i.e., self-handicap). This makes for a dynamic negotiation where children must assess, and if necessary, act upon the other child’s responses. Thus, RTP facilitates prosocial cooperation strategies, partially predicated on dominance and negotiation to maintain a harmonious relationship. This implies a give-and-take relationship where the attacker becomes the defender and *vice versa*.

Jarvis (2006) puts forth that RTP is an activity rooted in bio-evolution as evidenced by its use among animals and, to some degree, other mammalian species. For example, when rats are deprived of this sort of play fighting, a host of cognitive and emotional deficits have been observed (Jarvis, 2006). Much like Lilliard et al.’s (2013) arguments, Jarvis (2006) claims that RTP should not be viewed as a physical activity in which one exclusively seeks dominance over the other as the ultimate goal but as a collaborative activity that entails social learning.

Given the nature of play specifically, RTP has unsurprisingly been extensively studied in children. Among some factors, socioemotional competence has been argued to be a central outcome of RTP where infants engaging in RTP exhibit a more positive affect (Nakagawa and Sukigara, 2014). Indeed, a recent meta-analysis showed that father and child RTP practices were positively correlated to emotional competence and self-regulation (Stgeorge and Freeman, 2017). Additionally, it has been shown to reduce multiple dimensions of aggression. In their intervention, Carraro et al. (2014) deployed RTP as a structured play fighting activity in school, which lowered physical and verbal aggression, hostility, and anger (Carraro et al., 2014; Carraro and Gobbi, 2018). Considering the social dimension, Pellegrini (1988) puts this to the empirical test by observing elementary school children engaged in RTP in the playground. Pellegrini (1988) contended that RTP was associated with social competence. RTP subsequently led to games with rules, but only for popular children; children whose RTP instigations were rejected led to aggression instead.

However, recent research contradicts RTP’s educational and psychosocial contribution. Veiga et al. (2020) found instead that pre-schoolers who engaged more in RTP exhibited more physical aggression. Garcia et al. (2020) found that adolescents who exhibited more deviant behavior also reported more recent involvement in RTP activities. Other works have also confirmed the positive association between higher aggression and RTP (Paquette et al., 2003; Flanders et al., 2009). Smith and Boulton (1990) examined the ambivalent character of RTP. They argued that RTP could indeed exercise a set of social skills, but it can also be used for social manipulation, such as overt domination.

Additionally, the perceptions of RTP are dominated by a view that it is generally inappropriate. Most pre-school teachers consider RTP dangerous and negatively associated with physical and mental outcomes (Boyd, 1997), or they lack the knowledge of RTP in general (Peterson et al., 2018). Furthermore, Storli and Sandseter (2015) showed that games with the most restrictions placed on them by pre-school teachers were RTP in nature, further reinforcing the notion that RTP is considered immoral.

Importantly, RTP is not a binary activity (i.e., either you do it or not) independent of moderating factors. Instead, RTP can differ in quality. For example, in reviewing the theoretical foundations for RTP, Fletcher et al. (2013, p.5) contended that as:

*“In high-quality RTP, the father is attentive and playful, and he communicates enjoyment at the competition between the two of them. He is attuned to the child’s abilities and interests and can motivate the child to re-engage. The father succeeds in keeping a good balance between actively challenging the child, on the one hand, and ‘letting the child win’, on the other hand.”*

This then includes levels of RTP dominance and turn-taking, which have been shown to be predictive of aggression in children (Flanders et al., 2010; Anderson et al., 2017). This is an important basis for the paper; RTP is assumed to be of high quality when there is a balance between the parties involved. Additionally, it contains a challenging element that makes RTP risky and exciting but confined within a safe space. This is indicative of the significance of a supervising element in RTP. Finally, moderating factors in RTP bear connotations to the early findings of Pellegrini (1988) where social status affected the outcome of RTP attempts. Thus, RTP is dependent upon individual and contextually contingent factors.

In conclusion, RTP is a concept with a promising empirical basis concerning its psychosocial and emotional contributions, but some moderating factors must be addressed. It appears that RTP can contribute to developmental outcomes, but the literature generally agrees that this is contingent on high-quality RTP and other contextual factors. In this paper, we advance this thought and argue that RTP can be effectively moderated to accommodate positive experiences and outcomes under a set of contextual rules. By doing so, we acknowledge the importance of contextual factors, but we remain true to the core argument of the framework; the bodily contact and experiential experiences stemming from RTP and MA can be directly responsible for developmental outcomes.

### Why Martial Arts and RTP?

MA is an umbrella term for various disciplines, such as wrestling, boxing, judo, and karate. While most research on MA tends to focus on motor and performance aspects, there is a solid body of literature on MA’s promising sociopsychological contributions (e.g., Blomqvist Mickelsson, 2021). Another distinctive feature of MA, its explicit *budo* philosophy, emphasizing a range of virtuous characteristics. Thus, a cursory review on the characteristics of MA indicates its appropriateness for psychosocial development.

A common denominator for most MAs is its emphasis on two combatants that face each other in sparring. Sparring can be defined as an intentional physical interaction and struggle to overcome the opponent. This is grounded in skill development and not in a desire to hurt the counterpart. The sparring constitutes a consensual social and emotional interaction through physical altercation often supervised by experienced trainers. Thus, RTP naturally resides within MA practice. RTP can exist in other sports too, but its role is less significant compared to that in MA. This is the intrinsic feature of MA that is important from an RTP perspective. MA practice (sparring) needs two bodies that work with and against one another in direct physical contact. It is not a sub-part of the sport, such as tackling is in soccer, but instead constitutes the main activity. Despite the synergy in properties between RTP and MA, these concepts have only been vaguely and unsystematically linked to one another previously.

Considering that RTP is an activity that, in its natural form, occurs in everyday settings, such as a schoolyard, between siblings at home, etc., RTP is often an activity negotiated exclusively by the involved partners. Moreover, while RTP can effectively achieve positive outcomes, it is a vulnerable activity that nevertheless can produce the reverse outcome (e.g., humiliation and over-stepping boundaries; Smith and Boulton, 1990). Thus, MA presents itself as an appropriate space; by embedding RTP within a moderated ruleset, one can control such factors and work intentionally toward positive outcomes. This echoes the sentiment of Carraro et al. (2014), p.1304. In relation to RTP, they posited that as: “*Due to its peculiar physical and psychological features and its behavioral antecedents, play-fighting in structured and supervised settings may be an effective activity to promote social and emotional skills, which can in turn be helpful in preventing self-perceived aggression*.”

An important distinction is made here between *training* in MA and *competing* in MA. Playful elements of MA are best exhibited in training between two consenting partners in a moderated environment. On the contrary, a competitive environment entails performative aspects and a lack of contextual moderation that is important when implementing RTP as an activity (Carraro et al., 2014). The nature of competition and the ambitions of elite athletes entail a comprehensive discussion concerning the potential psychoeducational contribution outside of the paper’s scope.

RTP involvement declines with age, and its meaning changes as we grow (Pellegrini, 2002). This has implications in a MA context where all age groups exist. Intuitively, as we mature, we also engage less in physical behavior and become more aware of social norms and conventions concerning physical behavior (see Boyd, 1997). It is a rare sight to see two adults wrestle for the fun of it at social events. Naturally, MA facilitates a platform where such behavior is encouraged regardless of age. We also propose that RTP in MA is elevated by the societal context we live in today. It has been argued that sport is an outlet for excitement and risk-taking in contrast to the boredom of everyday life (Elias and Dunning, 1986). MA carries intrinsic connotations of risk, and more so than most other sports. Considering that children are more prone to physical play, the risk and physicality of MA would intuitively seem more salient to adults, hence why it may also be increasingly exciting for adults to engage in MA. Without further differentiation between the levels of enjoyment of MA between young people and adults, we propose that this perception or actual risk that MA carries should appeal across all ages. Importantly, this risk (perception) is moderated by MA coaches who have an important responsibility in making sure there is a balance between challenge, risk, and safety.

Just as RTP varies in its quality and outcomes, so does RTP in MA. Translating Fletcher et al.’s (2012) definition to a MA context, we suggest that the father-child dyadic example is re-defined by two individuals who engage in sparring. The sparring constitutes the challenge (i.e., the risky play) and excitement that is the foreground to RTP. However, much like the father controls the activity and switches back and forth between submissiveness and dominance, the sparring involves reading one another’s body language, emotional reactions, and responding appropriately. In short, this entails empathetic self-handicapping.

However, a clear reorganization of Fletcher et al.’s (2012) definition must be performed concerning the father and child relationship. This dyad has a strong, pre-determined social structure with biological connections that are difficult to transfer directly into a MA gym. We understand two sparring partners as two individuals not deterministically bound by a certain social structure or in a power position of some kind. That is, shaping risk-taking, challenge, and turn-taking are elements that both engage into a greater degree than fathers and sons. Nevertheless, it should be noted that self-handicapping will depend on a range of factors that may vary between practitioners. The most clear-cut example is that of physical size. A substantially physically heavier practitioner will need to engage in more self-handicapping and determine the pace, turn-taking, etc., to a greater degree than practitioners who are evenly physically matched. The ability (or responsibility) to shape the sparring properly thus exists on a spectrum mediated by factors, such as physical size, skill, and much more. This mediation becomes even more important, considering that many MAs adopt belt systems. A higher rank is the proof of acquired expertise and knowledge within a particular MA. This is usually not limited to skill acquisition but relates to general knowledge of the sport and its inherent principles. Here, higher-ranked members may therefore act as role models setting examples for beginners or members with lower grades. Setting these examples can include normative and ethical frameworks, which are important when engaging in sparring.

Importantly, our argument in this paper is that corporeal experiences through MA are responsible for psychosocial growth. This is not to be confused with a complete rejection of the significance of environmental factors. By now, it is well established that the ecology of sport participation is imperative for developmental outcomes (Coakley, 2011). The same holds for RTP. Our critique is rather that contemporary theories completely disregard the corporeal experience. Thus, we argue that we need to understand how corporeal experiences in sport also facilitate psychosocial growth in addition to contextual factors. In fact, these environmental factors must cultivate the corporeal experience. We define this as high-quality RTP cultivated within MA settings. This is contingent on individual characteristics, and (1) philosophy, (2) organizational culture and capacity, and (3) members in MA gyms. A healthy synergy between these levels cultivates a functional MA environment in which RTP becomes a natural element.

Thus, we also acknowledge the existence of MA environments that do not cultivate high-quality RTP. Here, principles that are important for RTP are violated, and these gyms often see a constant flow of members joining and quitting. This unsustainability can be caused by coach misconduct, hyper-competitive and insensitive sparring, and other factors that cause RTP to be less enjoyable.

Before discussing psychosocial MA research and outlining MA components necessary to achieve high-quality RTP, we must first address the critique that has remained central to MA practice.

#### Martial Arts as Aggressive and Deviant Forms of Leisure

The most profound critique of MA is that it constitutes a space where violence is glorified and aggressiveness is promoted. This argument is a misunderstanding based on invalid conceptions of MA, at times expressed in influential articles on the subject. For example, a benchmark study of power sports and MA revealed how MA was associated with more antisocial behavior (Endresen and Olweus, 2005). Thus, it has been argued that MA is amoral (Dixon, 2015) and constitutes an inappropriate space for young people to socialize. This idea is not a peculiar one; according to Bandura’s seminal social learning theory, aggression is cultivated by learning aggressive actions, which at first glance seem to be a core feature of MA.

This is indeed a peculiar paradox. Why would sanctioned violence be educative and promote psychosocial behavior? We argue that first and foremost, this critique is based on a straw man argument that asserts that there is little consensus between practitioners. Many critiques of MA (e.g., Dixon, 2015) seem to have made few attempts to make sense of MA and instead follow the opinion of outsiders or how the media depicts MA. The growing interest in media for competitive MA could contribute to the misinterpretation that there is a fundamental intention to inflict physical injuries within MA in general.

Importantly, the claim that MA constitutes a violent space with amoral connotations makes the implicit assumption visible, namely, that critics conflate general violence to what happens inside MA gyms. This calls for a reorganization of the idea of violence. According to the World Health Organization (WHO), violence is defined as:

“*The intentional use of physical force or power, threatened or actual, against oneself, another person, or against a group or community, that either results in or has a high likelihood of resulting in injury, death, psychological harm, maldevelopment or deprivation.”*

This definition must be viewed in parallel to the purpose of athletes in general, namely, to perform well. In a MA context, this translates into dominating your partner physically, which inevitably poses risks for injuries. Nevertheless, when injuries occur in MA, it is rarely the result of intention but rather an accident (Martínková et al., 2019) and is seen as undesirable (Abramson and Modzelewski, 2011; Channon, 2020). For example, the British Aikido Association explicitly states in their code of conduct that students have an obligation to protect each other’s health and wellbeing (Martínková et al., 2019). This complex balance is akin to the central concepts of RTP: to joyfully engage in physical contact to pursue dominance but never to harm.

In WHO’s definition, the intention of the action is central. The intention of everyday practice in MA training is never to cause injury. Therefore, WHO’s definition of violence is not appropriate when considering MA. Instead, we argue that MA can be viewed as a collaborative activity. Training in MA is entirely dependent upon partners. Consequently, one’s further development in MA depends on one’s training partners and having other practitioners who are regularly willing to engage in MA practice with oneself. This is likely not to be the case if one practitioner exerts violence as per the definition above by another practitioner. Considering this, most MA gyms have clear boundaries of how this “violence” is exerted. In our experiences, MA practitioners who cross these boundaries become stigmatized, are shown the door, and/or are generally frowned upon as they pose a risk to others.

#### Martial Arts Psychosocial Benefits

Early research noted that MA could function as an arena where physical exercise is not the only outcome. MA practitioners develop physical attributes, but they also challenge themselves mentally by engaging in sparring, thus developing specific mental attributes (Weiser et al., 1995). This is evident from many studies that consistently show that a long training experience within MA is positively correlated with a set of psychological factors. This includes better self-regulation (Lakes and Hoyt, 2004; Nakonechnyi and Galan, 2017), attentional control (Lakes et al., 2013), resilience (Greco, Cataldi and Fischetti, 2019), and lowered aggression (Nosanchuk and Lamarre, 2002; Harwood et al., 2017). Consequently, there is an array of psychosocial benefits stemming from MA practice, and we will focus on mainly four: aggression, self-regulation, compassion, and prosocial behavior. This is by no means an exhaustive account of each construct but instead serves to indicate MAs link to each of these.

Aggression is widely considered as undesirable and has implications for young people’s psychosocial development and adjustment. Most studies suggest that a longer time participating in MA is correlated with lower aggression (for reviews, see Harwood et al., 2017; Blomqvist Mickelsson, 2021; Lafuente et al., 2021). There is some longitudinal evidence to this too. Hortiguela et al. (2017) conducted a quasi-experiment comparing a MA group to a control group. Interestingly, the authors found that the MA group achieved better attitudes toward unjustified violence than the control group.

Most recently, Lafuente et al. (2021) performed a systematic review on the topic. They found that, specifically, *traditional* MAs were associated with decreases in aggressive traits. In addition, traditional MA is associated with a set of philosophical factors interrelated to organizational characteristics, which we will return to later. Further corroborating Lafuente et al.’s (2021) findings, Blomqvist Mickelsson (2021) reviewed all existing literature on one MA with traditional underpinnings. Again, the findings were consistent in that it was connected with lower levels of aggression and an inclination toward adequate psychosocial health.

Early research into the mechanisms behind MA’s mitigating effects on aggression suggested that MA practitioners learn to master their aggressiveness and emotional affect in a safe space (Twemlow et al., 2008). Twemlow et al. (2008) postulated that MA is an emotionally corrective experience where MA students can transform destructive aggressiveness into prosocial behavior. Other work suggested that practitioners may increase aggression when acquiring new techniques but lower aggression once they can self-regulate and control the technique (Nosanchuk and Lamarre, 2002). Ultimately, Nosanchuk and Lamarre (2002) note that aggression tends to be lowest at the black belt level (i.e., the practitioners with the most experience). These findings are partially consistent with our own experiences in that we have found that aggression in MA practice is not desirable. Aggressive and impulsive acts are rarely good in terms of performance in MA. In MA subcultures, fighting out of sheer rage is actually frowned upon (Abramson and Modzelewski, 2011). Instead, composure, calculation, and reflexiveness characterize what we have seen as the best performances. It is thus no coincidence that aggression may be prevalent in novel students (Daniels and Thornton, 1990; Blomqvist Mickelsson, 2020) but seems to vanish at higher levels. However, we expound on these early reflections to suggest that it is not merely about technical acquisition and mastering but also about the relationship to one’s training partners and how we nurture this relationship. This, in turn, entails self-regulation, compassion, and prosocial behavior.

Self-regulation can be viewed as the opposite of aggression. It is a limited mental resource, which when depleted has been seen to negatively correlate with an increased propensity for aggression (DeWall et al., 2007). It also has several implications for the development of youths. For example, the ability to better withstand instant gratification in children has been linked to more successful outcomes later in life (Mischel et al., 1988).

Characteristics of MA entail that one can endure mentally and physically draining training. In addition, MA practitioners are faced with the difficulties of sparring where willpower and skill are tested directly against another opponent. This calls for a more long-term self-regulatory ability (i.e., maintaining training) but also short-term regulation (i.e., keeping composure in intensive situations and not giving up).

Not surprisingly, MA seems to increase an individual’s self-regulation in general. The seminal work of Lakes and Hoyt (2004) can be considered the benchmark study for how MA can help youths in their self-regulation. In their school intervention, Lakes and Hoyt (2004) found that an informed taekwondo program significantly helped youths develop self-regulation, including how they reacted to challenging situations. This has been corroborated in various studies (e.g., Zivin et al., 2001; Blomqvist Mickelsson, 2021). For example, Zivin et al. (2001) found that youths involved in MA learned to breathe to control their impulses, which is consistent with MA practice – holding your breath or having the body control the mind is detrimental to MA performance. Considering long-term self-regulation, Massey et al. (2013) found self-regulation to be a critical factor in how MA practitioners prepared themselves before bouts.

Findings in adjacent fields of contemporary research provide nuanced insights into the mechanisms of MA and self-regulation. In sociological literature, several scholars have engaged in MA practice to understand it further. Drawing on a three-year ethnography, Sugden (2021) trained and competed in MA and found that it had great implications for mental health. Specifically, Sugden (2021) highlighted how students needed to endure training to acquire new techniques, test them, and engage in a trial-and-error process, which is heavily time-consuming. In addition, facing several setbacks as a novel student (e.g., losing to lesser opponents), MA entails developing resilience and self-regulation not only in the immediate situational context. Thus, one is faced with one’s ego in a very confrontational manner. This confrontational and demanding nature of MA has several consequences, one of which is that attrition is high (Sugden, 2021). The need to self-regulate and endure training is thus a consequence of MA training but can also be a significant barrier to sustained participation. Burrow (2014) suggested that this kind of ability has an impact beyond the gym. For example, enduring MA training and knowing that one could defend oneself can lead to a greater inclination to reject inappropriate suggestions even if the consequences are dangerous.

Finally, we turn to compassion and prosocial behavior. These are discussed simultaneously given their closely related nature. Weiser et al. (1995, p.120) too noted that MA provides a socializing and educational context: “*Mas [martial arts] training on values such as respect, humility, responsibility, perseverance, and honor. These attitudes and values become a model for the student, which can then be generalized to many arenas of living*.”

Twemlow et al. (2008) analyzed a MA bullying intervention in school. Not only did they find that MA participation significantly reduced aggressive tendencies and increased helpful behavior, but they also found that empathy could mediate these findings.

Contemporary research points to MA sparring in itself as a potential catalyst for producing empathy and prosocial behavior. Recently, it has been shown how sparring, even tough and semi-competitive sparring, can serve as the basis for friendship by developing respect for one another’s skill and willpower (Blomqvist Mickelsson, submitted). Indeed, according to Sugden (2021), the basis for prosocial encounters lies in conjunction with sparring. In the immediate aftermath of sparring, one is given the opportunity to reconcile and contemplate on the sparring with one’s partner. Considering the intimate and risky nature, other MA scholars have argued that MA training partners share an experience characterized by rawness and honesty (Green, 2011).

In turn, engaging in such risky activities may spur in-group bias and social bonding where members perceive themselves and one another as superior as they are able to partake in MA (Abramson and Modzelewski, 2011). Within such a sub-community, individuals need to engage in sparring with people from various backgrounds. Other research has shown that this acquisition of empathy and respect is transferred to everyday life (Chinkov and Holt, 2016).

In the neurophysiological domain, Rassovsky et al. (2019) examined oxytocin production in MA practitioners. Oxytocin is a peptide hormone responsible for the social behavior of mammalian species and has been shown to affect parent-child attachment and prosocial behavior. Oxytocin production has, for example, been shown to be prevalent in interactions between parent and child that involve physical touch (Feldman et al., 2010). Considering MA’s intimate nature, it is not surprising that engaging in MA training increased oxytocin production significantly (Rassovsky et al., 2019).

Importantly, the physical contact in MA is not a wild, blood-stained, and uncontrolled activity. Instead, it is two agents who respond and adapt to one another in a collaborative context with liminal space, defined by a common understanding of the activity (Kimmel and Rogler, 2019). These confrontations often end with an affective and ritual reconciliation embodied as a bow, a touch of gloves (Clapton and Hiskey, 2020), or hugs (Jones, 2001). In this sense, sparring is conceived of as a dyadic encounter that promotes prosocial behavior (Rassovsky et al., 2019). Individuals thus learn to perform within the context of a risky conflictual situation and to re-establish a healthy attitude toward a training partner once the sparring is over. According to Clapton and Hiskey (2020, p.3), “*Martial arts might be implicitly and explicitly entraining value-driven abilities to stay affiliatively engaged in conflict situations of high relational threat and end such conflicts by reaffiliating, with minimum harm done*.” Other research that supports this conclusion is Blomqvist Mickelsson (2020) who showed that MA practitioners in a 5-month intervention consistently increased levels of prosocial behavior. More importantly, while being an under-researched theme, it has also been shown how this ability to cope with conflict in MA affects how practitioners cope with relationships outside of the gym (Foster, 2015).

In conclusion, MA practitioners learn to respect and care for one another while simultaneously pushing each other’s mental and physical boundaries (Chinkov and Holt, 2016; Sugden, 2021). Practitioners not only care for each other out of respect for a partner’s physical health, but also to nurture the social relationships that exist on the mat. In other words, it is imperative to moderate one’s own physical strength and behavior if faced or training with a physically inferior training partner. Again, this is akin to a core tenet of RTP that emphasizes how play fighting is collaborative, and not intimidating or dominating. This is the core of RTP’s utility within the MA context. Physical contact is entangled in different ways. It entails cooperation, but at the same time, posing challenges for the other individual.

#### The Premises of Psychosocial Development in MA

So far, the outcomes and micro-level effects of RTP elements within MA have been discussed. We now turn to the contextual factors that make MA a suitable space for RTP. In summary, these can be placed in an ecological perspective, including philosophy, organization, and peers.

#### Philosophy

Few sports, if any, have such an explicated philosophy as MA. Emerging as an academic field of its own, MA philosophy has been extensively researched. The definition taken from SBMAF concerns the *budo spirit*, which emphasizes mindfulness, discipline, spirituality, and self-development. Budo is a Japanese term referring to a warrior’s code. This partially implies physical movement and has connotations of the military, but also peace and reconciliation. The main concept derived from budo can be interpreted as the road toward self-development, both physically and mentally. This is a pacifistic philosophy, focusing more on self-development than on instrumental victory. While this is an interplay (i.e., striving for victory and pushing one’s boundaries may lead to self-development), the focus is still on the former. This is a key addition to why MA may be viewed in the light of psychosocial development. Seminal sport sociologists have critiqued the “car-wash effect,” namely, that sport participation cleanses undesirable characteristics and personal defects (Coakley, 2011). Instead, one must pay attention to the ecology surrounding a specific sporting environment (Trulson, 1986), including its philosophy and axiology.

A seminal scholar on the topic, Wojciech Cynarski, suggests that the epistemology of MA is “… *as a psycho-physical path of practice, introversion and intuitive knowledge, and at the same time self-discovery by the individual in training*” (Cynarski, 2013, p.3). Accompanying this epistemology are the values adopted across most MAs, including respect, loyalty, humility, and so on (Martínková et al., 2019). However, there are also selected axio-normative systems that can shape the lifestyle and internalization of certain practitioner values. For example, Cynarski (2019) suggests that “classic” karate originating from Okinawa, Japan, is strongly influenced by the student-master relationship. This shapes “classic” karate practice in that, for example, contemporary karate schools in Okinawa accept relatively few students. Another example is how karatekas are socialized, with respect for the elders. According to Cynarski (2019), this can be understood as partially contingent on Confucianism traditions and is more salient in countries with stronger social hierarchies. Consequently, one must pay attention to social and cultural circumstances surrounding a particular MA (practitioner; Cynarski, 2013). In essence, understanding the philosophical foundation and axiology of one’s setting has pedagogical implications. Without understanding the systems of meanings, the coach remains an empty shell that merely delivers instructions and techniques.

Because MA philosophies impact on the code of conduct for MA practitioners, it is not surprising that Cynarski (2006) and Martínková et al. (2019) argue that without this ethical framework that permeates most MAs, there can also be negative consequences. This becomes even more important, considering that some categories of MA practitioners differ to some extent in how they adopt ethical considerations (e.g., Kostorz and Sas-Nowosielski, 2021). The seminal work of Nosanchuk and MacNeil (1989) can serve as a brief illustration of this. The authors examined two taekwondo groups; one group exercised without the traditional philosophy and *vice versa*. The results revealed that the group with the philosophy lowered levels of aggression, while the reverse was true for the counterparts.

The budo philosophy is more explicit within traditional MA compared to its modern counterpart. This typology is not without issues; however, there are distinctions. For example, traditional MAs have a clearer link to self-development outside of the physical domain. In addition, coaches, for example, will discuss mentality in MA explicitly and outline this in contrast to other modern competitively-oriented MAs. While this may be the case, our experience is that MA philosophies seem to exist as a generic, sometimes vaguely defined guiding star even in modern MA gyms. Thus, this aspect becomes important for actors within MA environments that pay little attention to this aspect. However, to our knowledge, this sort of philosophy is unparalleled in other sport that seems to be drawing from even broader generic statements and guidelines. Thus, the budo philosophy is part of a MA culture that nevertheless varies in strength across gyms.

#### Organization

An organization permeated by a healthy philosophy will also strive to maintain and reproduce this philosophy in an organizational and embodied form. We argue that this is imperative in a MA context, perhaps more so than in other sports. The reason is simple. The consequences are far worse in MA than in other sports if boundaries are overstepped.

Research primarily highlights the role of the coach. Accordingly, MA trainers are of great value as role models but also as moderators of sparring. As put forth by MA practitioners in Chinkov and Holt’s (2016) study, the trainers intentionally put practitioners in tricky and demanding situations. However, they make sure that they can cope with the demands placed upon them. This notion is reinforced by Sandford and Gill (2019), who interviewed several long-time MA coaches. These coaches consistently referred to matters, such as social expectations, physical experiences, and mindful training, but also highlighted the matter of matching practitioners equally. Drawing from Partikova (2019) study on MA coaches, it was highlighted how not only physical skills are taught, but also how values are transferred.

Aligning with the trainer’s scope in Chinkov and Holt (2016), Sandford and Gill’s (2019) participants also made visible the need for practitioners to engage in physical contact and to be in fighting situations in a non-malicious way. By engaging in this moderated but challenging situation, developmental outcomes may be achieved. Here, we can sense how coaches cultivate the previously mentioned risk of MA. The risk is constitutive of the excitement and as part of the challenge that MA sparring presents. Consequently, these findings highlight how MA contexts are indeed risky practices in which practitioners run the risk of being physically (and psychologically) humiliated if the training is poorly supervised. This calls for increased attention to organizational capacity and awareness where the coach is at the center of attention. Navigating and commanding this context adequately is thus crucial to avoid detrimental outcomes, and specifically, to intentionally be able to produce developmental outcomes.

The coach can be considered the bridge between philosophy/organizational culture and the members. Correct coaching and assessment are thus essential for high-quality RTP in MA.

#### Members

The final moderating factor presented here is that of peers. One can play soccer hard or unfairly, but one cannot unfairly engage in MA without more dire repercussions. Latching on to Clapton and Hiskey’s (2020) idea of MA as a cultivator for compassion, Haudenhuyse et al. (2012) also detected how MA practitioners needed to self-regulate when faced with lesser skilled training partners, clearly indicating the collaborative and nurturing role of MA. The socially and physically molded peers within a healthy gym environment will know boundaries and serve as gatekeepers when faced with bullies or “really” violent individuals. As such, the environment as a whole (i.e., philosophy, coach, and peers) can moderate how much one is allowed to dominate the other and cultivate a space for safe learning and RTP. Juxtaposed with the research on father-child RTP, Paquette (2004) claimed that a father could facilitate the learning process for the child through modeling and governing the play – therefore, the father in the current paper’s case is a mixture of all previously mentioned components. In a more abstract sense, the father is the philosophy that shapes the code of conduct, the organization, and coaches. In the coach-student dyad, the father is the coach who shows boundaries and how to train correctly. Between members, the role of the father does not exist in this sense. Instead, being cultivated by the factors above should make members inclined to shape the sparring properly, both presenting a challenge while simultaneously caring for the other individual.

Here, we highlight one example in the literature that already exists. MA scholar Spencer (2013) elucidated a case where a practitioner, perhaps unintentionally, bullied a physically lesser practitioner. By that time, Spencer himself was very familiar with the gym environment. Following the line of thought that one becomes socialized into the cultural norms of a MA gym, Spencer (2013) alerted the coach, and the latter restored order swiftly (and harshly). Considering the former section about how sparring is a collaborative activity, this is a striking example of what happens in a caring gym environment when boundaries are broken and collaboration becomes bullying. This is reflective of both author’s experiences as well – the organizational milieu will allow different degrees of domination to be exerted. This thus elucidates the importance of MA trainers being able to analyze, assess, and understand levels of intensity in sparring.

With this backdrop, one may ask as: Are discipline, humility, and respect for a fellow human exclusive to a MA gym or in sport at all? The answer is no, but in conjunction with the nature of RTP and the physical struggle between two combatants in MA, these factors become much more salient and important given the dire physical (and mental) consequences that can arise if sparring inside a MA gym overstep boundaries compared to other activities. We do not claim to present contextually unique factors; we claim to present a unique theoretical synergy between a fitting sport within a theoretical framework that must be surrounded by contextual factors already has mapped out by the literature.

#### Martial Arts as Fun Leisure

Finally, and central to RTP, MA is fun. Notwithstanding our own bias, we can relate this to the literature. In broader leisure research, one of the main motivations behind engaging in leisure activities is to have fun. This can be exemplified through Sugden (2021) description of his experience as close to obsessive, and his striking engagement in his fieldwork where he was immersed in a Brazilian jiu-jitsu gym as a complete novice but ultimately ended up competing. More importantly, Theeboom et al. (2009) explored children’s participation in MA, and a core finding was that children preferred to “kick and fight” (p.25) during practice, which served to amuse them. Accompanying this joy in engaging in free fighting was also the fact that “*According to the children, this kicking and fighting is not about hurting and inflicting damage on others*” (Theeboom et al., 2009, p.25). One can thus draw a direct parallel to the central tenet of RTP, namely, its emphasis on *pretend* playing where physical encounters are not intended to be physically intimidating or harmful but rather seen as a central part in having fun and negotiating social roles through one’s own body. Winkle and Ozmun (2003) pointed out that for MA to be (psychosocially) fruitful, the delivery needs to involve enjoyment and fun. This aligns well with the fact that enjoyment was a core pillar in a study by Hortiguela et al. (2017) who even operationalized a variable as “enjoyment” to capture this dimension. We believe that “fun” in MA intersects at the crossroads of risk and challenge that should be accommodated and “safetified” (Martínková and Parry, 2017) by the MA club.

**Figure 1:**
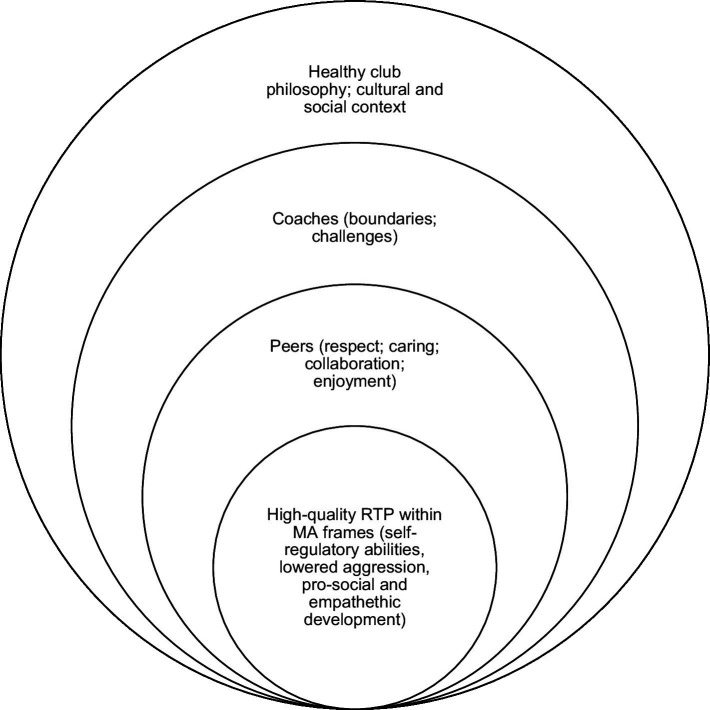
An ecological model of high-quality RTP within MA.

In short, people engage in and are willing to spend their free time on extracurricular activities for several reasons, but having fun is undoubtedly one of them. In this sense, MA is no different. The ecological model we propose can finally be roughly visualized in Figure 1, which displays how the experiential experience and process of RTP are embedded within the formal and informal frames of MA. These ultimately converge into what we call high-quality RTP. To summarize our outlined model, we suggest that RTP contributes toward a theoretical understanding of psychosocial development through the corporeal experience in MA. However, for high-quality RTP to occur within MA, one must consider the ecology in which MA is performed. The ecology of MA must cultivate high-quality RTP. An autoethnographic example of the first author can illustrate an ecology that did *not* cultivate high-quality RTP. This took place in a large-scale commercial mixed martial arts gym. While there was a strong sense of comradery, it was contingent on how one conformed under the training regime. In turn, this training regime was ultra-competitive. Sparring was always conceived as competition, i.e., the climate was goal oriented and not mastery oriented. This was mainly spurred by the head coach who set the tone for the existing climate. The consequences of this were many. First, there was slow technical learning progress for practitioners since few techniques were taught. This progress was also slowed down because losing in sparring was stigmatized. Thus, it was (socially) too risky to try new techniques, little task mastery was involved, and the stakes involved spilled over into taking on a social character. This ultra-competitive climate also gave rise to numerous injuries. That is, practitioners did not care for one another, meaning they failed to meet an essential criterion for high-quality RTP.

## Discussion

The paper has proposed a model in which RTP can be embedded within MA practices. Considering the positive but slightly ambivalent empirical support for RTP given its claim for reduced aggression, situating RTP practices within a moderated structured setting can elevate its potential to contribute to developmental outcomes. Taking the premise that the core values of MA are upheld, high-quality RTP can be practiced through MA.

The model serves as an analytical orientation for practitioners, for example, physical educators attempting to promote psychosocial change through sport. Notably, MA has already been advocated as an excellent addition to physical education curriculums (Winkle and Ozmun, 2003). However, the theoretical analysis of *how* MA contributes to psychosocial development has remained limited to pointing out the empirical patterns between MA participation and the psychosocial outcomes. MAs’ psychosocial black box is well captured in the article of Martin (2006) entitled as: *The psychosocial benefits of traditional martial arts training: What most instructors know but cannot articulate*. Here, Martin (2006) argues that it is difficult for the common coach to explain the success of anecdotal cases. Deficiencies in explaining how psychosocial development comes about are also prevalent in broader SFD research (Coalter and Taylor, 2010). By incorporating the concept of RTP into the framework, it addresses how the physical properties of MA contribute toward such outcomes. This makes the framework distinct from that of other theories. Drawing from our literature review, PYD is the most dominant. Yet, PYD places its focus on contextual factors almost exclusively and correlates them with internal traits. While PYD has proven to be a compelling theory in a host of domains, when implemented in the sport-for-change literature, it tells us nothing about the properties of sport in itself that can facilitate development.

Most importantly, the current model has implications for practice. First, it is directly applicable through SBMAF. This renders the current paper more practically viable than most others, considering its immediate tie to practice. The current model will inspire and shape future education for practitioners in the Swedish MA context. Secondly, it will allow coaches to address psychosocial change through MA explicitly. Most analytical accounts of how MA mitigates aggression, for example, are anecdotal and insufficient. Common laymen can claim that MA serves as an outlet for aggression, for example, therefore, practitioners do not subsequently feel the need to be aggressive in their everyday lives. We doubt that MA would be an enjoyable context if practitioners partook to alleviate aggression exclusively. Following Martin (2006) idea, we believe that many MA coaches partially understand the mechanisms behind MAs psychosocial effect but fail to articulate this or conflate it with other factors. By understanding RTP’s social and psychological effect, coaches are more well equipped to supervise and moderate training intentionally. Although it may be more of an organizational matter, it also has implications for members. Understanding the boundaries of RTP and the consequences of breaches may inform members’ understandings of sparring.

Although this model is positioned in developmental psychology, it is also of sociological interest. Considering how high-quality RTP in MA emphasizes empathy, prosocial behavior, and caring for one’s training partners, it is no surprise that other works have captured how MA environments are characterized by strong bonding elements in a more sociological spirit (Andreasson and Johansson, 2019). Thus, MA RTP has implications not only for oneself but also for the social connections and impact one makes through sport.

The current paper has put forth that *MAs* are appropriate forms when considering embedding RTP within a structured setting. However, it should be considered that psychosocial outcomes have varied between different sorts of MAs (Cynarski and Obodynski, 2004; Litwiniuk et al., 2009; Graczyk et al., 2010) and within the same MA (Korobeynikov et al., 2019). For example, boxers seemed to exhibit more aggressive behavior than jiu-jitsu and capoeira practitioners (Kusnierz et al., 2014). In particular, this sparked a debate in *Science*. Within this debate, it was argued that state of the art (MA) research showed little support for its psychosocial contributions (Mercer, 2011; Strayhorn and Strayhorn, 2011). Strayhorn and Strayhorn (2011, p.310) contended that as: “*Martial arts training is a heterogeneous independent variable with average effects that may be negligible or even negative*.” We suggest that this is only partially true. MA cannot be viewed as a panacea to social illnesses without considering specific characteristics, hence our outlined model. As shown in contemporary MA research, failing to make accurate distinctions between MAs (or other important features) will inevitably reduce the quality of subsequent analysis (Blomqvist Mickelsson, 2021; Lafuente et al., 2021). Here, the generic philosophy that surrounds traditional MA is promising. Nevertheless, it is not automatically granted but needs to be scrutinized. As Cynarski (2019) has already shown, despite promising MA philosophies, the meaning of MA is not static across time or practitioners. Consequently, it remains imperative for coaches to understand the embedded context in which MA is performed for pedagogical purposes. One still needs to pay attention to the cultural and social factors embedded within specific MAs or gyms. In addition, from an RTP perspective, coaches need to understand the developmental trajectory of individuals and how this temporal aspect may give MA shifting meanings for individuals.

The current framework has been narrowed down to the sport domain and further to the sub-domain of MA. The idea here is to offer a detailed and practitioner-oriented approach that aligns well with specific sports. To this end, we identified what we believe is a sport with strong connotations to the physical and playful nature of RTP but that also consists of the moderating factors that empirically have been seen to impact the outcomes in RTP practice. This is not to say that RTP is incompatible with other contact sports, but the moderating factors of such sports should be considered if developmental outcomes are to be achieved.

One limitation should be addressed here. The model has refrained from discussing competition as a salient mechanism for sustaining interest and enjoyment in MA. While some authors have proposed that competitive MA functions as a mutual risk construction built on collaboration and consensus (Channon, 2020), we have refrained from discussing competitive MA for two reasons. This paper attempts to address MA as a psychosocial tool for the general practitioner/audience (laymen, if you will). In our experience, very few that engage in MA ever compete in it. Going from practice to competition seems to be characterized by a higher threshold compared to other sports. In comparison, it is almost impossible *not* to compete if involved in organized soccer. It seems as if in MA, training is the goal in itself for most practitioners.

Secondly, RTP does not translate well into an actual competition because of its emphasis on *turn-taking*. While sparring can be shaped and, to some degree, subject to turn-taking, MA competition logically does not allow for this. Consequently, we believe that this is a reasonable limitation. However, this is not to say that competitive aspects do not sustain the interest of MA practitioners. On the contrary, such aspects undoubtedly play a crucial factor here too.

One final consideration should be made. MA is an arena dominated by males. This is much in line with RTP research where primarily boys are examined and especially the father-son dyadic relationship. While this is a clear limitation concerning the model’s applicability for females, it is yet another synergy between RTP and MA. Consequently, the model may elucidate how RTP is shaped, experienced, and understood by primary males in MA.

## Data Availability Statement

The original contributions presented in the study are included in the article/supplementary material, and further inquiries can be directed to the corresponding author.

## Author Contributions

TBM and PS have contributed toward the conceptualization and reviewed and edited the final manuscript. TBM drafted the manuscript with frequent input from PS. All authors contributed to the article and approved the submitted version.

## Conflict of Interest

The authors declare that the research was conducted in the absence of any commercial or financial relationships that could be construed as a potential conflict of interest.

## Publisher’s Note

All claims expressed in this article are solely those of the authors and do not necessarily represent those of their affiliated organizations, or those of the publisher, the editors and the reviewers. Any product that may be evaluated in this article, or claim that may be made by its manufacturer, is not guaranteed or endorsed by the publisher.
